# Novel Coronavirus (SARS-CoV-2) in Water and Environment—A Scoping Review

**DOI:** 10.3390/life12040520

**Published:** 2022-03-31

**Authors:** Taufique Warsi, Tanvi Arora, Syed Shams Rizvi, Ali Raza Moosvi, M. A. Mohammed Aslam, Mohammad Muqtada Ali Khan, Arifullah Mohammed

**Affiliations:** 1 Electrical Geophysics Division, CSIR-National Geophysical Research Institute, Uppal Road, Hyderabad 500007, India; 2 WOTR Centre for Resilience Studies (W-CReS), Watershed Organisation Trust (WOTR), Pune 411009, India; 3 School of Earth Sciences, Central University of Karnataka, Kalburgi 585367, India; shamsrizvi@cuk.ac.in (S.S.R.); moosvi1@cuk.ac.in (A.R.M.); maslam@cuk.ac.in (M.A.M.A.); 4 Department of Geoscience, Faculty of Earth Science, Universiti Malaysia Kelantan, Campus Jeli, Locked Bag No. 100, Jeli 17600, Kelantan, Malaysia; muqtada@umk.edu.my; 5 Department of Agriculture Sciences, Faculty of Agro-Based Industry, Universiti Malaysia Kelantan, Campus Jeli, Locked Bag No. 100, Jeli 17600, Kelantan, Malaysia; aurifullah@umk.edu.my

**Keywords:** SARS-CoV-2, pandemic, economy, climate, SDGs, mitigation, COVID recycling

## Abstract

A pneumonia outbreak was primarily reported in the fall of 2019 in Wuhan, Hubei province, China, with the identity SARS-CoV-2, a novel coronavirus. It quickly grew from a local epidemic to a global pandemic and was declared a public health emergency by the WHO. A total of three prominent waves were identified across the globe, with a slight temporal variability as per the geographical locations, and has impacted several sectors which connect the world. By March 2022, the coronavirus had infected 444.12 million people and claimed 6.01 million human lives worldwide, and these numbers have not yet stabilized. Our paper enlightens readers on the seven strains of human coronaviruses, with special emphasis on the three severe deadliest outbreaks (SARS-2002, MERS-2012, and COVID-19). This work attempts a comprehensive understanding of the coronavirus and its impact on the possible sectors that link the world through the economic chain, climate conditions, SDGs, recycling of the event, and mitigations. There are many points that are raised by the authors in the possible sectors, which are emerging or are as yet unnoticed and thus have not been taken into consideration. This comprehension will leave sets of new challenges and opportunities for the researchers in various streams, especially in earth sciences. Science-integrated research may help to prevent upcoming disasters as a by-product of (existing) epidemics in the form of coronavirus.

## 1. Coronavirus: A Brief History

June Almedia, a virologist who left school at the age of 16, came into the limelight recently when the century’s deadliest pandemic hit the globe with the identity of SARS-CoV-2. This virus was caused by the first type of coronavirus, which was identified by her, way back in 1964 in St Thomas’s hospital in London, where she worked then. Collaborating with Dr David Tyrrell, who was renowned in the common cold research field, this discovery was published in *British Medical Journal* in 1965 [1,2,3]. Tyrrell was not able to grow the agent in tissue culture back then, but Hamre and Procknow worked on the viruses, and they succeeded in growing it [4].

**Figure 1:**
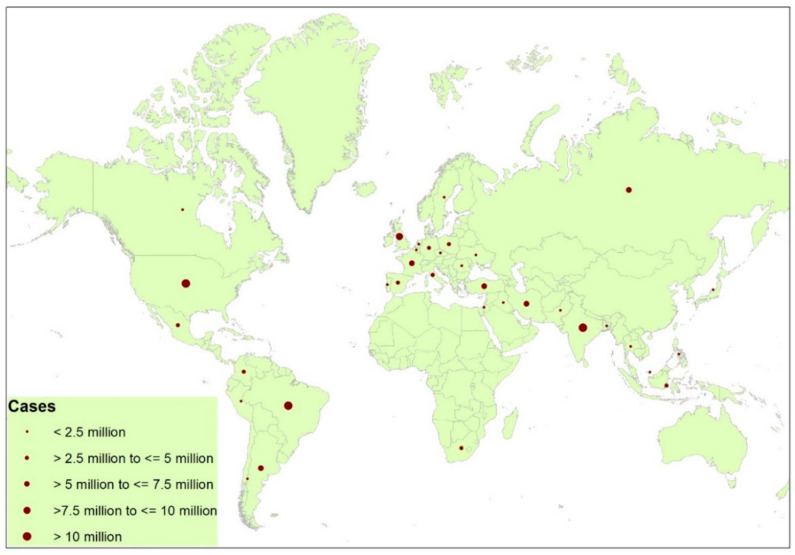
Worldwide distribution of corona cases across the spectrum (above 5000 cases, as of 26 September 2021).

Zoonotic pathogens have been found to be responsible for most of the viral outbreaks in the past and are most likely to be so in the future too. Some of these pathogens are potentially strong and reach the advanced transmission stage to make it more capable for human-to-human transmission, but some of them do not. As per a study, prior intimation was given by Fan et al. that three coronavirus outbreaks took place in the past two decades, and another may again occur in near future; his review is based on the domain knowledge on viral diversity, reservoir hosts, and the geographical distribution of bat coronaviruses [5]. The poor understanding of viruses across the globe led this epidemic to flourish globally which gave rise to the biggest pandemic of this century (Figure 1). It is believed that the very first victim of 2019-nCoV was a 55-year-old individual from Hubei province in China, a case which dated back to 17 November 2019, as per the news in the *South Morning China Post*.

It is perhaps time to agree that the virus was completely of natural origin, although at the beginning of this outbreak, it was considered to be an intentional outbreak. In March, a study came out that denied these theories and reported that the virus had some unusual features very similar to the endangered mammal pangolin, which suggests the possibility of this mammal being the intermediate carrier for this virus. However, further study is required to conclusively prove this statement [6,7].

It currently appears very difficult, if not impossible, to develop a comprehensive appreciation of the impact of the virus on the lifestyles and livelihoods of human beings in the world as we know it today. As time passes, the case fatality rate is in ascent, in opposition to the earlier estimation, and the fatality rate can fluctuate through the virus time frame, which is unknown. The novelty of the disease and the contribution of the super spreaders make it more complex for containment of the virus. Ignorance of early warnings and the lack of preparedness drag down the escaping capability; in turn, it impacts the whole travel and tourism sectors, which has blown away the economic stability of the globe. Footprints of the virus may be reactivated as weather, fickle through time, may cause a rejuvenation in the cycle and recycling or by dragging the tail of the corona breakthrough curve.

## 2. Human Coronaviruses

Four groups of genera are classified for coronaviruses, i.e., Alpha, Beta, Gama, and Delta. Among these, Alpha and Beta capture mammals, whereas Gamma and Delta are responsible for birds. There are seven known segments of human coronaviruses (Table 1), out of which four are found to be of mild symptoms similar to the common cold, and three are respectively severe in nature [8,9].

In the initial phase of the outbreak, Chinese authorities identified an individual with pneumonia hospitalized in Wuhan. Afterwards, authorities precluded SARS-CoV, MERS-CoV, influenza, avian influenza, adenovirus, and the rest of the common respiratory pathogens [10,11]. Initially, the name given to this pandemic by World Health Organization (WHO) was 2019-nCoV, but in the later phase of surveillance it was renamed as SARS-CoV-2 by the International Committee on Taxonomy of Viruses. There is a significant risk factor in coronaviruses that varies widely at its bar. Death rate varies broadly from 0 to 30% in the common cold and MERS-CoV, respectively. Symptoms of SARS-CoV-2 were found to be cough, high fever, and shortness of breath. Its transmission is from person to person through droplets of cough and sneeze from the infected source. It can be potentially severe, and in some cases, it can claim human life [12].

### 2.1. Taxonomy and Classification

Coronavirus belongs to the vast group of viruses, from under the family Coronaviridae with the subfamily of Ortocoronavirinae found in the order Nidovirales and realm Riboviria. The crown-like viral particle enveloping CoVs lends it its name: coronavirus. On the way to RNA synthesis or host response modulation, the involvement of polymerase (RdRp) and other non-structural proteins are enumerated [13,14]. The diameter of the virion in coronaviruses is enumerated as approximately 120 nm, with the surface projection of club-shaped protein. There are four canonical structural proteins found in coronaviruses: the outer biggest transmembrane spike protein (S), followed by small envelope protein (E), membrane (M), and nucleocapsid (N) and some accessory proteins [15].

### 2.2. Novelty of SARS-CoV-2 in Contrast with Earlier Outbreak

In the wake of rapid transmission, the last two decades were dominated by major zoonotic coronaviruses that claimed thousands of human lives. In 2002, typical pneumonia was reported called severe acute respiratory syndrome (SARS), which marked the first pandemic of the 21st century, with its origin and epicenter in Southern China (Guangdong). It started on 16 November 2002 and ended in July 2003; its incubation period was 2 to 7 days, which was measured to be less effective in comparison with the rest of the viruses. It spread to approximately 30 countries including China, with 8422 reported cases and fatalities around 916, which promptly bothered the WHO enough to announce it as a worldwide health threat [16]. During the SARS outbreak, it was considered that the virus-host was the bat and that it had the ability to jump over another, which mutated in such a way that it could infect another intermediate host, probably civet [17]. The intermediate host, later on, made all the differences and mutated to humans [16,18,19].

A decade later, it happened again. Now the epicenter of the virus found a new spot in Saudi Arabia, which spread its wings in the peak period of March 2014 and folded back by the end of 2015. The very first case of Middle East respiratory syndrome (MERS-CoV) was reported in Saudi Arabia in June 2012. From then till October 2018, 2260 confirmed cases were reported, with 803 fatalities. The cases flourished in 27 countries, with Saudi Arabia alone contributing 71% of cases [20]. At the emergence of the SARS-CoV-2 outbreak, the case fatality rate (CFR) was higher (approximately 35%) because of its novelty and lack of preparedness, but as the time passed, the decline in CFR was marked, which was controlled mainly by vaccination and herd immunity, and it came down to 1.35%. Its fatality rate was comparably higher than the rest of the viruses, which was estimated at around 35%, and the bat reservoir had enough capability to mutate to the animal reservoir (Table 2), with the result being that it has been found that it mutated to the camel as an intermediate host [21,22].

The emergence of a novel coronavirus SARS-CoV-2 brings back the images of SARS-CoV from two decades ago—a déjà vu—as if we were looking into a rear-view mirror. In 2019 during the nCoV outbreak, it was being estimated that the bat reservoir mutated enough and directly jumped into humans, but in some studies, it has been enumerated that the bat virus initially mutated to angolin and then passed on to humans [6]. The basic reproduction number was calculated in Wuhan and in the Diamond Princess cruise to understand the passage of virus with the help of the exponential growth model method, and it was found to be in the range of 2.24–3.58 [22,24]. This was certainly not the end, and changes took place in the genetic sequence, which gave rise to mutation. Subsequently, differences in the genetic sequence came out with several variants. The WHO has listed variants of concern (VOC) that emerged from the outbreak; Alpha, Beta, Gamma, Delta, and Omicron were the most prominent ones that triggered the major waves across the globe [16,25,26].

## 3. Revolutionizing Epidemic to Pandemic

The first two phases of distribution were relatively marginal as the cases started emerging from one to another. The reproductive number (Ro) for the COVID cases was found to be in the range of 1–3; later on, there was a debate in the scientific community with regard to the fluctuation of range.

**Figure 2:**
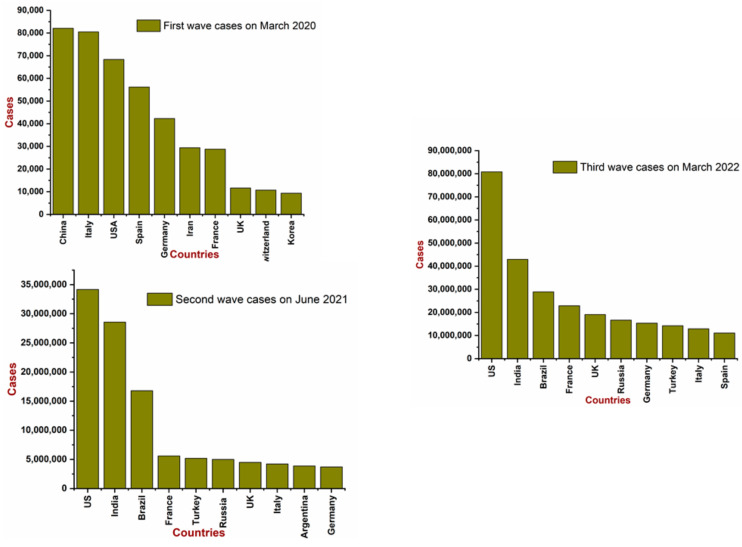
Migration of COVID-19 across the globe (Data source: Worldometer and WHO).

The novel strain of the coronavirus was considered to have originated from the fish market or wet market of Hubei province; since the virus was novel, the people were not aware of the gravity of this pandemic. The disease was initially found to be of virus-induced pneumonia symptoms as per the report of clinicians, with the additional symptoms of rising body temperature and depletion in the lymphocytes number and white blood cells [27]. In the initial phase of the outbreak, Japan and Thailand were registering their presence in the death marathon, and suddenly Diamond Princess started raising its hand, and South Korea came into the picture. Now, one could see the panicked faces of other countries. It was certain that no one was prepared enough to tackle the situation, which was worsening day by day. The prime concern about this virus was the incubation period (3–14 days), which usually occurs before the symptoms start to begin. Still, the majority of the population were busy in solving the conspiracy puzzle of whether it was a manufactured virus in the laboratory or a natural one, rather than being busy in solving how to contain it. On 11 March, the WHO announced this outbreak as a COVID-19 global pandemic. During the first wave, China was exasperated by trying to contain the virus, and it was recorded as the first country with the maximum number of active cases. However, from the second wave onwards to the present, the US overtook the race with a massive margin and even now the US holds the first position in the chart (Figure 2).

So far, India and South Korea were praised by the rest of the world for containing the virus through their proactive approaches. India had taken an early decision of country lockdown, keeping in view the vulnerable health care system and the vast population, because social distancing and preventive measures seem to be very hazy in these circumstances. On the other side, African countries were so far untouched by this pandemic, but they were in a worrying situation because of a healthcare system unprepared to cope with the coronavirus pandemic. With an 11 million population, the nation of South Sudan has five vice presidents and four ventilators; 10 African countries do not have a single ventilator [28,29].

## 4. Concussion of the Pandemic

The impact of COVID-19 on the global economy is maximum at its scale; still, after-shock impacts are yet to be revealed once the virus is contained globally. This period will witness the consequences in the time to come. Major worrisome points are discussed here.

### 4.1. Economic Indicators

The outbreak left the century’s financial crisis of 2007–2008 and 2002–2003 far behind and built its castle in the pandemic territory. The market for crude oil hit the lowest in history during the tenure of the pandemic. The world is acknowledging that life is more important than oil. People are on the road everywhere, even during the safest measures taken by world leaders. Worldwide jobs are affected, daily to medium businesses are at a loss, gender inequality is more evident in keeping the economy running, agriculture farming is suffering, and daily wagers are crying. People are looking to governments across every part of the globe to keep them alive and healthy, over being safe.

**Figure 3:**
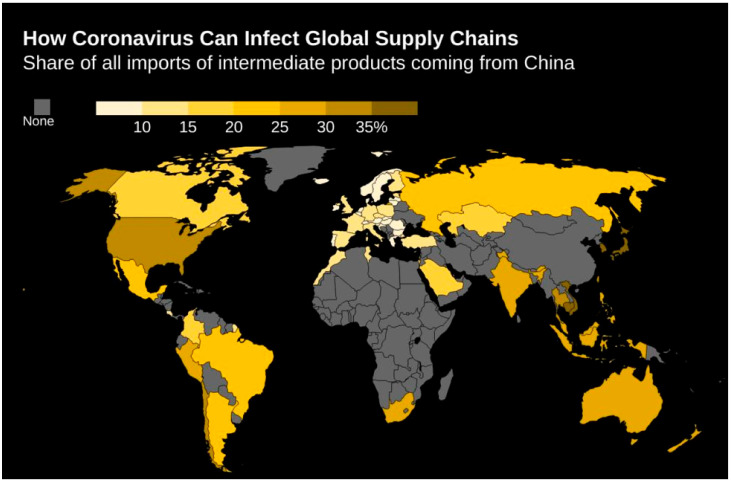
Impact on the global supply chain given the coronavirus outbreak, Cousin (2020). (Source: OECD TiVA Bloomberg Economics, Adapted with permission from Ref. [32]).

Any interruptions in China’s economy will likely have repercussions on the rest of the global supply chain, as the global economy is pretty much interconnected with China, which is the core manufacturing hub of several global business actions. The global economy was already advancing slowly; meanwhile, the virus outbreak held it down, indicating a global recession for the year [29,30,31]. COVID-19 has impacted the Chinese manufacturing and services sectors, entrenching a record fall in January–February. Automobile sales fell by 80%, whereas exports slipped by 17.2%; this was the signal of economic chain failure and was well justified by the decrease in pollution level and shipping traffic. As a result, this impact on the economy and GDP of China dragged other countries into the arena that were closely connected, such as Europe, Japan, and the US. To contain COVID-19, measures were taken, such as quarantines, travel bans, and closure of public places, which are directly going to impact supply and demand. Since China plays a vital role in the global supply chain and contributes as the largest exporter of intermediate manufactured products, so the impact on the economy of China and country lockdown will lead us to global supply chain disruptions (Figure 3).

As per the calculation of OECD trade data, almost 20% of global imported products were contributed by China in 2015 [32]. At the annual spring meeting of the International Monetary Fund (IMF) and the World Bank, IMF chief Kristalina Georgieva expressed the tension posed by coronavirus. She said the world economy was already crawling when the pandemic jumped on its back, which in this scenario may come result in the biggest recession of the century (IMF report; 18 April 2020). With hopes of a strengthening global economy, people were expecting good times ahead, but the year 2022 let them down comprehensively and recorded a half of a percent decline for 2022 over late 2021 (IMF report, 18 April 2020). Now, the policymakers of the countries of the world had to play a vital role in getting rid of this long-lasting economically laden pandemic. Policymakers must still target their help toward the most vulnerable households and smaller firms by waiving through several kinds of security and bills.

### 4.2. Environmental Resistance

History clarifies the consequences of past pandemics and its by-products, which turned to be positive in many of the cases. However, nobody wanted to see this kind of environmental benefit coming along with such tremendous human suffering. China and Italy experienced a drastic improvement and positive change in air quality since the lockdown. On normal days, Venice’s Grand canal was flooded with boats and its resultant pollution—the water now is running clear, and there is clear sight of fishes, whilst the dolphins are even returning without the boats! A downturn in the curve of asthma, cancer, and lung and heart diseases does not happen frequently, and the climate is no more causing deaths [33]. In response to the sudden shutdown across the country, economic activity and the use of fossil fuels reduced drastically in China alone, contributing to a 25% fall in carbon emissions while containing the virus (which is estimated to be 200 million tons of carbon dioxide, probably half the annual emissions of Britain) [34]. The pace at which the environmental changes are occurring implies the emergence of new and various pandemic diseases in future. Wet markets are the main source of these diseases that ultimately contaminate the air, groundwater, and wastewater. The animals decompose after their death, and these viruses accumulate in the soil, which reaches the underlying rocks and eventually reaches groundwater. Many researchers have started IoT-based detection techniques to identify levels of infection in groundwater [35]. This family of viruses can remain infectious for long periods in water and pasteurized settled sewage, which indirectly points out that the contaminated water or the water that is disposed after washing hands is a potential surrogate source of virus and thereby a threat in the future.

### 4.3. Vindication and Deterrence of New Strategies

In the initial phase of outbreak, vaccination for SARS-CoV-2 was in its embryonic stage, and scientists across the globe worked hard for a single cause, came out with an efficient vaccination, and more than 70% of the population are presently fully vaccinated. However, some scientific and experimental studies came out that claimed to be able to contain the virus to some extent. As per the study of past contagious epidemics and pandemics, one can take away the lesson of the importance of breaking the chain, which must be of prime concern till vaccination and special therapeutics are in the market. Therefore, we had to get back to the classical public health measures for limiting the pandemic’s further acceleration; thus, initially there were some preventive courses viz. social distancing, isolation, quarantine, herd immunity, raising immunity levels, restricting the usage of right hands, and community containment which were to be implemented on a massive scale and which are continuing in most of the countries [35]. SARS-CoV and MERS-CoV are the perfect examples of taking a lesson on in how to disrupt the chain and restrict it from further spreading [17,36].

Since the outbreak, various effective vaccines have been available to the public at-large, with consideration of variable age limits, number of doses, booster drugs, different health conditions, etc. Due to the unpredictable evolution of the virus, the use and trials of various drugs has been restricted. The use of drugs initiated with the use of hydroxychloroquine has drawn massive attention for the treatment of COVID-19 patients. Hence, 30 COVID-19 patients were taken with the consent of the Shanghai Public Health Clinical Centre and divided into an HCQ group and a control group to observe the comparative impact of HCQ. As a result, the prognosis was found to be good in the patients, and a large-scale sample study was suggested to provide more specific guidance on HCQ treatment [37,38]. Since nanotechnology already flourished in all the possible sectors of science—from the oil industry to water science—one of the most recent works shows the efficiency of novel carbon quantum dots emerging as a water tracer for groundwater studies. At the same time, one study came out that imparted our idea about the use of carbon quantum dots as anticoronavirus therapy; seven different CQDs were synthesized and used for the treatment of HCoV. Surprisingly, an equally large and restrained process was seen at the viral replication step [39,40]. In another study, an increase of a unit degree Celsius and a unit percent in relative humidity could lead to a decrease in viral reproductive number by 0.0383 and 0.0224, respectively. Further, the drugs were moderated according to the increase in temperature and humidity conditions, speculating that the depletions of the influenza transmission even in the summer and rainy season in the northern hemisphere were considered to be blessings to contain the virus. However, spatial and temporal variation also plays a vital role in the varying intensity of influenza. [41,42]. Health researchers put many old nonspecific antiviral drugs, broad-spectrum antiviral drugs, antiretroviral drugs, antimalarial drugs, antibiotics and antiparasitics, nonspecific anti-inflammatory and immunosuppressive drugs, kinase inhibitors, monoclonal antibodies, hormonal preparations, cardiovascular drugs, blood and blood-forming organs, and various supplements to use in different capacities, along with new discoveries. Data on the treatment of the viral infection are now in use and available from various databases, where an umpteen number of drugs are mentioned [25,43].

## 5. Vigilance towards Recovering from Corona in the Near Future

At this juncture, the most frightening nightmare may not be a new family of viruses but the invisible threat from the same family, peeping through the rear-view mirror. Multiple factors give vital information on a temporal basis and cannot be ignored while fighting with such a deadly pandemic. There are some points that are of real concern, mentioned as follows:

- Gender: As per the data, cases of COVID-19 are equally distributed in both genders [44]. However, some studies report that men are more susceptible to this virus as compared with women [45]. In many countries, the ratio is 3:1 of getting infected till death (WHO 2002). Still, this inference was based on initial datasets and needs to be better established.
- Age: The most affected age group is >30 according to the diagnosed cases in Hubei province [46]. The cases can vary continent-wise as per the geography, so country-wide surveillance is required to recognize the patients who need specific treatment. Initially, it was reported that children were least affected by COVID, but recent cases of children infected in Telangana are redefining these theories also. Even kids in the age group of infants to 10 years are prone to get an infection. During the evolution of the pandemic over the past 3 months, the infected age group was redefined and needs to be better established.
- Environment: Biomedical waste is affecting all of the environment. Wastewater testing can be an early identification of the virus if it returns. The samples tested in the United States, Netherlands, and Sweden have shown the presence of RNA traces of the virus [47]. Such study has not been taken up in most of the countries so far. In a study that is in progress in the Netherlands, sewage data are to be compared with patient data and the correlation is to be obtained by the researchers. Studies have revealed that the virus can appear in feces within three days of infection, long before the person develops the symptoms of the virus. This can be an early indication of the infection caused in the patient. Additionally, research is ongoing for its possible transmission through feces [48].
- Animals and other creatures: A new study suggests that stray dogs are prone to COVID and that they act as intermediate hosts towards its spread. Another study mentions that COVID-19 can infect cats and dogs, though it is still not clear whether felines can spread the virus to people. However, these data are based on a few incidents [49].
- Climate: Coronavirus can remain active on surfaces from hours to days according to a study published in *The New England Journal of Medicine* [50]. As per the WHO, the coronavirus can be deactivated by exposure to a temperature of 60 °C for 45 min. However, many experts also believe that SARS-CoV-2 is seasonal, which could imply that outbreaks of COVID-19 cases will occur in winter.
- Waste management: Hand sanitization bottles are inflammable and need to be safely disposed of. Landfills in India are already struggling with unwanted pollution, and most of them are non-sanitary landfills. This plastic waste is going to add to the landfill heaps and thereby lead to environmental pollution. Many countries do not appear to have a strategy for dealing with such crises currently, even after the battle against COVID-19 is over. India has a major livelihood dependency on manual scavengers employed in almost 18 north Indian states. On 16 February 2020, *The Hindu* has reported that despite the 2013 legislation on employment as manual scavengers, there are at least 48,345 people employed in this job. Are we ready to risk the lives of 48,345 families, amongst million others, in the biggest democracy of the world?
- Water cycle: As per the NITI Aayog (2018) report, 166 million people in urban slums and rural areas of India have no access to clean water, and millions of them lack proper sanitation. The same report mentions that around 2,000,000 people in India die every year due to water-borne diseases caused by contamination. It is well documented by researchers that if the water cycle becomes aerosolized (by SARS-CoV), it could expose a large number of people to infection, even with quarantine measures to isolate infected individuals. Commercial, residential, and hospital water or sewer water contaminated with persistent infectious viruses may affect other individuals, even they have been removed from the place. The threat with these viruses stands tall and high.
- Fecal Transmission: A recent study in *The Lancet* points out the possibility of extended duration of virus in feces and which can stay up to 5 weeks after the patient’s respiratory samples have tested negative for SARS-CoV-2 RNA [51]. Open defecation, especially in Indian villages, is always a challenge. Thus, this may add up to the risk of recycling the virus into the system. Additionally, the WHO mentions a case study of finding COVID-19 in a community through the leakage in sewage pipes of toilets.

## 6. Impact of COVID-19 on SDGs

**Figure 4:**
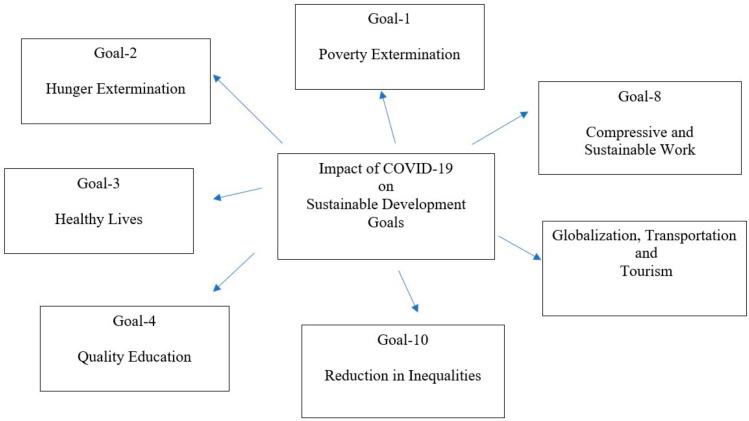
Impact of COVID-19 on SDGs (Jude and Adaeze 2020). (Source: www.eresearchjournal.com, accessed on 15 December 2020. Adapted with permission from Ref. [52]. Jude Chukwunyere Iwuoha and Adaeze Ukamaka Jude-Iwuoha (2020)).

In April 2020, most of the countries have implemented a massive lockdown to save the lives of their people, which has a great influence on the Sustainable Development Goals established by the United Nation in 2015. These seventeen goals were established to promote a better life and supportable future for all the people of the globe by 2030. The SDGs most affected by COVID-19 were goal-1, goal-2, goal-3, goal-4, goal-8, and goal-10 (Figure 4) [52]. During the lockdown periods, the hunger and poverty rate, education cycle were affected, there were job losses, and the healthy life and economy of nations were distressed [52,53]. The 2030 agenda for SDGs includes a common idea of peace and prosperity for the globe. The key areas of SDGs contain five pillars, such as people, prosperity, planet, peace, and partnerships, and governments under these main classes distribute budgets for different industries to achieve their goals (Wikipedia, Ankita et. al. 2020). The COVID-19 has gotten in the way of this achievement of goals, and in every individual in the countries it has touched, it has brought a distressing social, economic, and political crisis that will leave a deep scar [54]. COVID-19 has exposed that globalization’s contagion risk is high in whatever form—economic, health, financial, trade, etc. [52]. Though the battle with COVID-19 is not over yet, the planet is still fighting with this pandemic and trying to re-establish global prosperity. In this regard, it is important to concentrate on the coexistence of biodiversity conservation and the development of human society via conferences and seminars with various equity needs [55].

## 7. Discussion and Remarks

This review paper was written keeping the current public health emergency in mind, with an attempt to make stakeholders aware of the consequences of this pandemic in as many aspects and sectors as possible and possibly offer researchers from other fields opportunities to contribute scientifically to their respective fields for this noble cause. It is hoped that this review will bring a lucid understanding of the major strain of coronavirus and will develop a better insight for future pandemics. SARS-CoV-2 is almost 2 years old in this world. There is a lot of unknown things to be explored, vetted, and established. The world’s leading epidemiologists believe that this cycle of existence and destruction of this virus may take a long process till vaccination stabilizes and percolates and many people become immune to it, yet there may be multiple unexpected outbreaks of the disease—some may be known, but many may be unknown. Contamination of fecal material in water is well known after past experiences, which directly challenges human health. Water can act as a vehicle for spreading viruses that can cause great damage to human society. The determination of the survival of human coronaviruses was carried out for filtered and unfiltered tap water, and it was found that temperature plays a vital role in the activation of viruses. It implies that a higher level of temperature of around 23 °C can inactivate the virus in 10 days, whereas in the state of 4 °C temperature it can last for more than 100 days [55]. In consequence, winter tides can bring more challenges and complexities, especially in high latitude countries. In the Indian system, the season and temperature will play an important role in the containment of the virus. Considering the disturbance in the natural/environmental cycles and repeated outbreaks, the government has to take multi-tasking measures.

These additional few questions may lead to new directions in our approach to this contagion:

- Spraying disinfectants over a large area, and repeatedly, may cause environmental pollution, which is dangerous for its climate change effects. Do we have a quantification of the damage done at the ground level? How are we going to neutralize this effect—because major data are lost and very few researchers are successful in capturing the local data?
- As a principle of the hydrological cycle, whatever goes in, comes out. Thus, what is the fate of this virus? How long it will remain in the water cycle in different forms? Is it possible to eradicate it from the atmosphere?
- How does fecal transmission of viruses (especially in India) contribute to contaminating the soil and what is the critical zone?
- The fumes and evaporation coming out of the soil may have a lifespan. Is there a possibility of any indirect suspicious transmission?
- Are aerosols formed out of COVID life-threatening?
- Once absorbed into the system, what can be the maximum life for this and can it be diagnosed with carbon quantum dots (CQD)?
- COVID-19 and climate change both are equally important challenges to be faced by human beings globally. There are good air quality days in China, and as per China’s Ministry of Ecology and Environment, there was an increase of 21.5% in good quality days in February in 2020 as compared with 2019. Satellite images released by NASA and ESA show a dramatic reduction in nitrogen dioxide emission in major Chinese cities between January and February. The cloud of toxic gases, once visible, had disappeared. Will we have more or less the same impact on the Indian environment system?
- Climate change and the role of water have no common strategy for coping with sudden and aggressive natural threats. The changes in the climatic pattern or water cycle are likely to have degrading or destructive effects on natural habitats, ecological systems, and man-made agricultural and irrigation patterns, in addition to the prevailing hydrological cycle. The effects may be serious and need a detailed analysis.
- Why is recurrence happening again, will it continue, and if so, how many times in the near future?

## Figures and Tables

**Table 1:** Classification of Human coronavirus at its critical bar.

Type (Coronaviruses)	Symptoms	Classified
1	Mild	Human coronavirus OC43 (HCoV-OC43)
2	Mild	Human coronavirus HKU1
3	Mild	Human coronavirus NL63 (HCoV-NL63, New Haven coronavirus)
4	Mild	Human coronavirus 229E (HCoV-229E)
5	Severe	The Middle East respiratory syndrome-related coronavirus (MERS-CoV)
6	Severe	Severe acute respiratory syndrome coronavirus (SARS-CoV or “SARS-classic”)
7	Severe	Severe acute respiratory syndrome coronavirus 2 (SARS-CoV-2)

**Table 2:** Illustrates the distinction among recent bat-borne coronaviruses.

Viruses	Source	Mediator	Acceptor	Case Fatality Rate	Incubation Period	Reference
SARS (2002)	Bat	Civet	Human	916/8422 × 100 = 10.8%	2–7 days	[19,21]
MERS (2012)	Bat	Camel	Human	850/2500 × 100 = 34%	2–14 days	[17,23]
COVID (2019)	Bat	Pangolin	Human	6.1 M/444.12 M × 100 = 1.35% †	2–14 days (still uncertain)	[6,7]

(† Case fatality rate is calculated as of 5 March 2022, with the available data provided by coronavirus world meter; these figures may vary as the containment time increases).

## References

[1] Tyrrell D.A., Bynoe M.L. (1966). Cultivation of viruses from a high proportion of patients with colds. Lancet.

[2] Almeida J.D., Tyrrell D.A.J. (1967). The morphology of three previously uncharacterized human respiratory viruses that grow in organ culture. J. Gen. Virol..

[3] Brocklehurst S. The Woman Who Discovered the First Coronavirus. https://www.bbc.com/news/uk-scotland-52278716.

[4] Hamre D., Procknow J.J. (1966). A new virus isolated from the human respiratory tract. Proc. Soc. Exp. Biol. Med..

[5] Li H., Mendelsohn E., Zong C., Zhang W., Hagan E., Wang N., Li S., Yan H., Huang H., Zhu G. (2019). Human-animal interactions and bat coronavirus spillover potential among rural residents in Southern China. Biosaf. Health.

[6] Andersen K.G., Rambaut A., Lipkin W.I. (2020). The proximal origin of SARS-CoV-2. Nat. Med..

[7] Saey T.H. No, the Coronavirus Wasn’t Made in a Lab. A Genetic Analysis Shows It’s from Nature. https://www.sciencenews.org/article/coronavirus-covid-19-not-human-made-lab-genetic-analysis-nature.

[8] De Groot R.J., Baker S.C., Baric R., Enjuanes L., Gorbalenya A.E., Holmes K.V., Perlman S., Poon L., Rottier P.J.M., Talbot P.J. (2012). Family coronaviridae. Virus Taxon..

[9] Wertheim J.O., Chu D.K., Peiris J.S., Pond S.L.K., Poon L.L. (2013). A case for the ancient origin of coronaviruses. J. Virol..

[10] Chan K.H., Poon L.L., Cheng V.C.C., Guan Y., Hung I.F.N., Kong J., Yam L.Y., Seto W.H., Yuen K.Y., Peiris J.S.M. (2004). Detection of SARS coronavirus in patients with suspected SARS. Emerg. Infect. Dis..

[11] Li X., Luk H.K.H., Lau S.K.P., Woo P.C.Y. (2019). Human Coronaviruses: General Features. Ref. Modul. Biomed. Sci..

[12] Sauer L.M. What Is Coronavirus?. https://www.hopkinsmedicine.org/health/conditions-and-diseases/coronavirus.

[13] Fan Y., Zhao K., Shi Z.L., Zhou P. (2019). Bat Coronaviruses in China. Viruses.

[14] Coronavirinae in ViralZone. https://viralzone.expasy.org/785.

[15] Fisher D., Heymann D. (2020). Q&A: The novel coronavirus outbreak causing COVID-19. BMC Med..

[16] World Health Organization Summary of Probable SARS Cases by Onset of Illness from 1 November 2002 to 31 July 2003. http://www.who.int/scr/sars/country/table2004_04_21/en/print.html.

[17] Chan J.F., Lau S.K., To K.K., Cheng V.C., Woo P.C., Yuen K.Y. (2015). Middle East respiratory syndrome coronavirus: Another zoonotic betacoronavirus causing SARS-like disease. Clin. Microbiol. Rev..

[18] Hon E., Li A., Nelson E.A.S., Leung C.W., Cherry J.D., Feigin R.D., Cherry J.D., Demmler G.J., Kaplan S. (2003). Severe acute respiratory syndrome (SARS). Textbook of Pediatric Infectious Diseases.

[19] Huang Y. (2004). The SARS epidemic and its aftermath in China: A political perspective. Learning from SARS: Preparing for the Next Disease Outbreak.

[20] Bleibtreu A., Bertine M., Bertin C., Houhou-Fidouh N., Visseaux B. (2020). Focus on Middle East respiratory syndrome coronavirus (MERS-CoV). Med. Mal. Infect..

[21] Petrosillo N., Viceconte G., Ergonul O., Ippolito G., Petersen E. (2020). COVID-19, SARS and MERS: Are they closely related?. Clin. Microbiol. Infect..

[22] Zhao S., Lin Q., Ran J., Musa S.S., Yang G., Wang W., Lou Y., Gao D., Yang L., He D. (2020). Preliminary estimation of the basic reproduction number of novel coronavirus (2019-nCoV) in China, from 2019 to 2020: A data-driven analysis in the early phase of the outbreak. Int. J. Infect. Dis..

[23] Memish Z.A., Mishra N., Olival K.J., Fagbo S.F., Kapoor V., Epstein J.H., AlHakeem R., Durosinloun A., Al Asmari M., Islam A. (2013). Middle East respiratory syndrome coronavirus in bats, Saudi Arabia. Emerg. Infect. Dis..

[24] Zhang S., Diao M., Yu W., Pei L., Lin Z., Chen D. (2020). Estimation of the reproductive number of novel coronavirus (COVID-19) and the probable outbreak size on the Diamond Princess cruise ship: A data-driven analysis. Int. J. Infect. Dis..

[25] Matta S., Rajpal S., Chopra K.K., Arora V.K. (2021). COVID-19 vaccines and new mutant strains impacting the pandemic. Indian J. Tuberc..

[26] Vasireddy D., Vanaparthy R., Mohan G., Malayala S.V., Atluri P. (2021). Review of COVID-19 variants and COVID-19 vaccine efficacy: What the clinician should know?. J. Clin. Med. Res..

[27] Zhou P., Yang X.L., Wang X.G., Hu B., Zhang L., Zhang W., Si H.R., Zhu Y., Li B., Huang C.L. (2020). A pneumonia outbreak associated with a new coronavirus of probable bat origin. Nature.

[28] Maclean R., Marks S. 10 African Countries Have No Ventilators. That’s Only Part of the Problem. https://www.nytimes.com/2020/04/18/world/africa/africa-coronavirus-ventilators.html.

[29] Musmar F. The Effect of Coronavirus on the Global Economy. https://besacenter.org/perspectives-papers/coronavirus-global-economy/.

[30] Hayat R. Economic Implications of the Coronavirus. RaboResearch—Economic Research. https://economics.rabobank.com/publications/2020/january/economic-implications-of-the-coronavirus/.

[31] Ivanov D. (2020). Predicting the impacts of epidemic outbreaks on global supply chains: A simulation-based analysis on the coronavirus outbreak (COVID-19/SARS-CoV-2) case. Transp. Res. Part E Logist. Transp. Rev..

[32] Cousin M. How the Coronavirus Can Infect Global Supply Chains. https://www.bloomberg.com/news/articles/2020-01-31/how-the-coronavirus-can-infect-global-supply-chains-map.

[33] Crist M. What the Coronavirus Means for Climate Change. https://www.nytimes.com/2020/03/27/opinion/sunday/coronavirus-climate-change.html.

[34] Ray P.P., Majumdar P. (2020). Coronavirus: A novel threat and ICT-based mitigation. Curr. Sci..

[35] Wilder-Smith A., Freedman D.O. (2020). Isolation, quarantine, social distancing and community containment: Pivotal role for old-style public health measures in the novel coronavirus (2019-nCoV) outbreak. J. Travel Med..

[36] Harapan H., Itoh N., Yufika A., Winardi W., Keam S., Te H., Megawati D., Hayati Z., Wagner A.L., Mudatsir M. (2020). Coronavirus disease (COVID-19): A literature review. J. Infect. Public Health.

[37] Chen J., Liu D., Liu L., Liu P., Xu Q., Xia L., Ling Y., Huang D., Song S., Zhang D. (2020). A pilot study of hydroxychloroquine in treatment of patients with common coronavirus disease-19 (COVID-19). J. Zhejiang Univ. (Med. Sci.).

[38] Singh A.K., Singh A., Shaikh A., Singh R., Misra A. (2020). Chloroquine and hydroxychloroquine in the treatment of COVID-19 with or without diabetes: A systematic search and a narrative review with a special reference to India and other developing countries. Diabetes & Metabolic Syndrome: Clin. Res. Rev..

[39] Warsi T., Bhattacharjee L., Thangamani S., Jat S.K., Mohanta K., Bhattacharjee R.R., Ramaswamy R., Manikyamba C., Rao T.V. (2020). Emergence of robust carbon quantum dots as nano-tracer for groundwater studies. Diam. Relat. Mater..

[40] Łoczechin A., Seron K., Barras A., Giovanelli E., Belouzard S., Chen Y.T., Metzler-Nolte N., Boukherroub R., Dubuisson J., Szunerits S. (2019). Functional Carbon Quantum Dots as Medical Countermeasures to Human Coronavirus. ACS Appl. Mater. Interfaces.

[41] Dalziel B.D., Kissler S., Gog J.R., Viboud C., Bjørnstad O.N., Metcalf C.J.E., Grenfell B.T. (2018). Urbanization and humidity shape the intensity of influenza epidemics in US cities. Science.

[42] Wang J., Tang K., Feng K., Lv W. (2020). High temperature and high humidity reduce the transmission of COVID-19. SSRN Electron. J..

[43] Sarkar C., Mondal M., Torequl Islam M., Martorell M., Docea A.O., Maroyi A., Calina D. (2020). Potential therapeutic options for COVID-19: Current status, challenges, and future perspectives. Front. Pharmacol..

[44] Wenham C., Smith J., Morgan R. (2020). COVID-19: The gendered impacts of the outbreak. Lancet.

[45] Chen N., Zhou M., Dong X., Qu J., Gong F., Han Y., Qiu Y., Wang J., Liu Y., Wei Y. (2020). Epidemiological and clinical characteristics of 99 cases of 2019 novel coronavirus pneumonia in Wuhan, China: A descriptive study. Lancet.

[46] Surveillances V. (2020). The epidemiological characteristics of an outbreak of 2019 novel coronavirus diseases (COVID-19)—China, 2020. China CDC Wkly..

[47] Mallapaty S. (2020). How sewage could reveal true scale of coronavirus outbreak. Nature.

[48] Saplakoglu Y. New Coronavirus May Spread through Poop. 20 February 2020. https://www.livescience.com/coronavirus-covid-19-spread-through-feces.html.

[49] Shi J., Wen Z., Zhong G., Yang H., Wang C., Huang B., Liu R., He X., Shuai L., Sun Z. (2020). Susceptibility of ferrets, cats, dogs, and other domesticated animals to SARS–coronavirus 2. Science.

[50] Van Doremalen N., Bushmaker T., Morris D.H., Holbrook M.G., Gamble A., Williamson B.N., Tamin A., Harcourt J.L., Thornburg N.J., Gerber S.I. (2020). Aerosol and surface stability of SARS-CoV-2 as compared with SARS-CoV-1. N. Engl. J. Med..

[51] Wu Y., Guo C., Tang L., Hong Z., Zhou J., Dong X., Yin H., Xiao Q., Tang Y., Qu X. (2020). Prolonged presence of SARS-CoV-2 viral RNA in faecal samples. Lancet Gastroenterol. Hepatol..

[52] Iwuoha J.C., Jude-Iwuoha A.U. (2020). Covid-19: Challenge to SDG and Globalization, Electronic Research. Int. J. Humanit. Soc. Sci..

[53] Roorda N., Corcoran P.B., Weakland J.P. (2012). Fundamentals of Sustainable Development.

[54] Srivastava A., Sharma R.K., Suresh A. (2020). Impact of Covid-19 on Sustainable Development Goals. Int. J. Adv. Sci..

[55] La Rosa G., Bonadonna L., Lucentini L., Kenmoe S., Suffredini E. (2020). Coronavirus in water environments: Occurrence, persistence and concentration methods—A scoping review. Water Res..

